# Effects of Methimazole vs Propylthiouracil in Newborns: A Comparative Review

**DOI:** 10.7759/cureus.41505

**Published:** 2023-07-07

**Authors:** Mehak Bhagat, Purnima Singh, Sindhu Meghana Sunkara, Merin T Abraham, Maria Jimena Barroso Alverde, Sravya R Mundla, Andrea Mizrahi Drijanski, Anna Jobilal, Mohit Lakkimsetti, Nandini Nair, Waleed Razzaq, Zain U Abdin, Ishita Gupta

**Affiliations:** 1 Internal Medicine, Government Medical College, Amritsar, IND; 2 College of Medicine, Gulf Medical University, Ajman, ARE; 3 Internal Medicine, Caribbean Medical University, Curacao, CAN; 4 Internal Medicine, Kasturba Medical College, Manipal, IND; 5 Internal Medicine, Anahuac University, Mexico City, MEX; 6 Medicine, Apollo Institute of Medical Sciences and Research, Hyderabad, IND; 7 Internal Medicine, Sri Ramaswamy Memorial Medical College Hospital and Research Center, Chennai, IND; 8 Internal Medicine, Mamata Medical College, Khammam, IND; 9 Internal Medicine, Byramjee Jeejeebhoy Government Medical College, Pune, IND; 10 Internal Medicine, Services Hospital Lahore, Lahore, PAK; 11 Medicine, District Head Quarter Hospital, Faisalabad, PAK; 12 Medicine, Dr. Rajendra Prasad Government Medical College, Tanda, IND

**Keywords:** fetal harm, birth defects, aplasia cutis, graves disease, teratogen, propylthiouracil, methimazole, hyperthyroidism

## Abstract

Hyperthyroidism is more common in women and the sensitivity of thyroid function changes during pregnancy. Excess levels of thyroid hormones and thioamides have a major impact on maternal and fetal outcomes. Our aim was to perform an extensive literature review and provide relevant details concerning the analytical and clinical aspects of the potential effects of the two main drugs used (methimazole and propylthiouracil) in newborns. A thorough literature review was conducted using PubMed and Google Scholar databases. In total, 10 relevant studies were identified and data from these studies were extracted and then extrapolated into results after analysis. Three out of four studies that used methimazole and carbimazole, one and two, respectively, showed adverse fetal outcomes requiring surgical management for congenital anomalies like aplasia cutis, patent vitellointestinal duct, and gastroschisis. Out of the three studies that used propylthiouracil, one baby underwent surgery for bilateral pyelectasis, vesicovaginal fistula, anal stenosis, and polydactyly. The findings of the aforementioned studies provide enough evidence to imply that the use of methimazole and carbimazole to treat antenatal hyperthyroidism has worse fetal outcomes than the use of propylthiouracil. Also, given the paucity of data in the existing literature regarding propylthiouracil’s effects on newborns, further studies in this demographic are needed.

## Introduction and background

Hyperthyroidism

Hyperthyroidism is a condition in which the thyroid gland is producing an excess of thyroid hormones. Thyroid hormones are needed for the body to create its own homeostasis because they help control the growth, development, and metabolic regulation of all the cells in the human body. When the thyroid is producing too much of them, they accelerate the metabolism, using a lot of energy and breaking the body’s homeostasis [1]. 

Comorbidities, age, and the amount of excess hormones can be factors that affect the way this pathology is clinically presented. Some signs and symptoms are anxiety, weight loss, sweating, polydipsia, dyspnea, tachycardia, and fatigue [2].

Hyperthyroidism can be caused by different pathologies such as Graves’ disease, adenoma, or a toxic nodule. These conditions change the way the thyroid regulates itself, generating an excess of thyroid hormones. There are other conditions that are temporary, such as thyroiditis and lymphocytic postpartum, which occur in 5-10% of women after giving birth [3]. 

To make the diagnosis, the clinical features must be explained with laboratory exams. Measuring the thyroid-stimulating hormone (TSH) and the free t4 levels should guide us to where the problem originated. If the TSH levels are low, but the free T4 levels are high, then we should think the problem is in the thyroid, therefore it would be primary hyperthyroidism. If the TSH levels are elevated and the T4 hormone is also elevated, then the problem probably comes from the pituitary gland. If the doctor suspects Graves’ disease, then anti-thyroid antibodies will be elevated. Subclinical hyperthyroidism can also be present, which would present with low TSH levels and normal free T3 and T4 hormone levels [3].

Treatment is usually given depending on the clinical symptoms and the severity of the pathology. Thionamide drugs (methimazole [MMI] and carbimazole) will reduce the formation of thyroid hormones. This has proven to work in 90% of patients. Other medications can be given to help with the symptoms, such as beta blockers and glucocorticoids. In patients that have Graves’ disease, radioactive iodine usually works, but it has many side effects. In other cases, thyroidectomy is indicated to eradicate the cause [3,4].

Hyperthyroidism in pregnancy

Subclinical hyperthyroidism in pregnancy is relatively uncommon and affects two in 1000 pregnancies. Overt hyperthyroidism is associated with an increased risk of spontaneous abortions, congestive heart failure, thyroid storm, preterm birth, pre-eclampsia, and fetal growth restriction [5].

The most common cause of hyperthyroidism in pregnancy is Graves’ Disease. IgG type of autoantibodies produced in the mother cross the placenta, bind to the thyrotropin receptor of the fetus, and cause birth defects like growth restriction, preterm birth, low birth weight, and fetal loss. Among anti-thyroid drugs (ATDs), propylthiouracil (PTU) has been used more than MMI in pregnant women. Various studies suggest that even a low therapeutic dose of MMI is more teratogenic than PTU and can cause aplasia cutis, choanal atresia, omphalocele, and tracheoesophageal fistula formation [6].

In a study conducted by Robin P. Peeters and colleagues from Erasmus Medical Center in Rotterdam, it was found that the risk of hypertension is increased in women with hyperthyroidism and that of pre-eclampsia is tripled in women who have high T4 and low TSH levels. They also reported that high levels of thyroid hormone are associated with an increased risk of endothelial cell dysfunction, leading to its activation, which forms an important basis for the pathophysiology of hypertension in pregnant women [7].

The most bizarre complication that can occur in a mother due to hyperthyroidism is thyroid storm, which is 10x more common during pregnancy and is characterized by high temperature (38 to 40°C), tachycardia (120-160 bpm), sweating, nausea, and irritability, which can progress to delirium, lethargy, and coma, if not treated promptly. It can be precipitated by infection, surgery, induction of labor, diabetic ketoacidosis, or even by anesthetic drugs used during c-sections. It is most common when hyperthyroidism is undiagnosed. Therefore, good antenatal care and timely screening are very important in all pregnant women [8].

Epidemiology 

Hyperthyroidism is more common in women than men, with a 5:1 ratio. The approximate prevalence of hyperthyroidism varies from 0.2% to 1.3% in parts of the world with sufficient iodine intake, increasing to 4-5% in older women, and an increase in smokers. Europe has a prevalence of 0.7% similar to the 0.5% prevalence in the USA. In 2016, Australia had a 0.3% prevalence. In general, the incidence of hyperthyroidism depends on the contribution of iodine to the diet of the population. There are higher rates of hyperthyroidism in countries with iodine deficiency [9].

There is a very high prevalence of alterations in thyroid function in women, with 3/1,000 having hyperthyroidism. Many thyroid symptoms begin at childbearing age, helping us relate pregnancy with thyroid diseases. The incidence of hyperthyroidism in pregnancy is low, and it ranges from 0.1% to 0.4% of all pregnancies (0.6% overt and 1.8% subclinical hyperthyroidism) [10,11].

Hyperthyroidism in pregnancy at this age is mostly caused by Graves' disease with an incidence of 55-80 cases per 100,000 per year in women >30 years, in women aged 20-29 years the incidence is 35-50 cases per 100,000 per year, and for women <20 years the risk is lower. The prevalence of new-onset Graves’ disease in pregnant women is estimated to be 0.05%, and the incidence of Graves' hyperthyroidism decreases in the second and third trimesters, after an increase in the first trimester. Graves' disease accounts for 95% of cases of hyperthyroidism during pregnancy. Another common cause is human chorionic gonadotropin (hCG)-mediated hyperthyroidism seen in 1-3% of pregnant women, and the prevalence of thyroid nodules in pregnancy has been reported to be 29% depending on the iodine status of the population. In areas with dietary iodine deficiency, another cause of hyperthyroidism in pregnancy is excessive thyroid hormone production by autonomous thyroid nodules, uncommon in women <40 years [12-14].

Methimazole 

MMI is a thionamide class of anti-thyroid drug and is widely used for treating hyperthyroidism due to Graves’ disease and toxic nodular goiter. It also helps achieve a euthyroid state before thyroidectomy for toxic multinodular goiter or toxic adenoma and ameliorates any hyperthyroidism symptoms that may occur during the procedure. It is also FDA-approved for the above purpose before radioactive iodine therapy [15]. 

MMI directly inhibits thyroid hormone synthesis by blocking thyroid peroxidase enzyme and subsequent iodination of tyrosyl residues on thyroglobulin. This finally reduces T4 and T3 production in the thyroid gland. However, it does not affect the levels of circulating or stored thyroid hormones. It also does not affect exogenous thyroid hormones [16]. 

MMI is easily absorbed via the gastrointestinal (GI) tract. It is metabolized by the liver and excreted in the urine. It has a half-life of approximately 12 hours [17]. 

Traces of MMI have been found in breast milk and it is known to cross placental tissue. For breastfeeding women, it is advised to regularly check the infant for potential adverse effects like blood dyscrasia. To minimize the infants’ exposure to the medicine, a 3-4-hour gap can be maintained between consumption of the drug and breastfeeding [18].

For antenatal use, the mother should be counseled on the potential risk to the fetus or alternative drugs should be used [19].

A common side effect seen with MMI use is developing an itchy rash. Severe effects include agranulocytosis, hepatotoxicity, hypothyroidism, and teratogenicities like aplasia cutis, choanal atresia, craniofacial defects, and esophageal atresia [20-22].

Propylthiouracil

PTU is a thiourea-derivative class of ATD. It is used as an alternative to MMI intolerance in Graves’ disease and toxic multinodular goiter, and to alleviate symptoms of hyperthyroidism prior to thyroidectomy or radioactive iodine therapy. It is preferred over MMI to manage hyperthyroidism in the first trimester of pregnancy because of the lower risk of teratogenicity and thyroid storm/thyrotoxic crisis because of its quality to inhibit the peripheral conversion of T4 to T3. PTU inhibits the synthesis of thyroid hormone by blocking thyroid peroxidase enzyme and subsequent iodination of tyrosyl residues on thyroglobulin. It also inhibits peripheral conversion of T4 to T3 [23,24].

PTU is easily absorbed via the GI tract with a bioavailability of 75%, metabolized by the liver, and excreted in the urine. It has a half-life of 1-2 hours. It readily crosses placental tissue and traces have been found in human breast milk [25].

PTU is found to be highly hepatotoxic in both adult and pediatric patients leading to acute hepatitis and liver failure. Performing liver function tests before initiating the drug is advised. Doctors should always look out for signs of liver injury, especially during the first six months of initiating the drug. Another grave side effect is agranulocytosis, especially within three months of commencing the drug. Hence, a complete blood count with differentials is performed before prescribing the drug. Doctors should always look out for signs of infections. Other adverse effects include hypothyroidism, rash, rarely congenital abnormalities, and antineutrophil cytoplasmic antibody (ANCA)-associated vasculopathy [20,26,27].

Our review

During pregnancy, various physiological changes occur that alter the thyroid hormone levels circulating in the maternal system. Multiple studies have established the sensitivity of thyroid function and levels during pregnancy. If hyperthyroidism in the fetus remains untreated or uncontrolled, it can lead to a range of negative consequences, such as premature delivery, restricted fetal growth within the womb, and distress experienced by the fetus. Therefore, to achieve favorable outcomes for the mother and the fetus, it is critical to assess and address thyroid dysfunction in pregnant women. Our aim was to perform an extensive literature review and provide relevant details concerning the analytical and clinical aspects of the potential effects of the two main drugs used (MMI and PTU) in newborns.

## Review

Methodology

Search Strategy

A literature review was performed using PubMed, Clinicaltrials.gov, and Google Scholar from the end of April 2023 to mid-June 2023 for articles on hyperthyroidism treated during pregnancy with either MMI or PTU. Only case reports were included in the search strategies. The search strategy excluded systemic reviews, cohort studies, meta-analyses, randomized controlled trials, clinical trials, observational studies, and reviews. Keywords and terms used for the search included were “hyperthyroidism”, “pregnancy”, hyperthyroidism in pregnancy”, “thyrotoxicosis”, “fetal outcomes in pregnant women with hyperthyroidism”, “Graves disease”, “adverse fetal outcomes”, “thyroid function in pregnant women,” TSH in pregnancy”, “antithyroid drugs”, “methimazole”, “propylthiouracil” (PTU), “hCG in pregnant women”,” congenital hyperthyroidism”, “ methimazole vs PTU”, and “fetal thyroid function”. 

Our peers evaluated all of the obtained results that met the inclusion period and criteria. Certain filters were applied such as publication date, language, and study population. We were able to narrow down our search by Boolean operators (AND, OR, NOT), citation chaining, and snowballing techniques that only reported adult patients, and recent relevant studies. The quality and credibility of the articles were evaluated by considering factors such as study design and methodology. Two authors independently searched for articles and two other authors independently screened articles. All disagreements were resolved via a senior author. A total of 10 studies from the PubMed database were identified. 

Data Screening

We only included articles that were published case reports, articles in English only, studies including only human data, and studies including pregnant women with hyperthyroidism before or during pregnancy. We included studies with pregnant women undergoing treatment using MMI or PTU for hyperthyroidism. 

We excluded articles, systematic reviews, meta-analyses, cohort studies, randomized controlled trials (open-labeled, double-blinded, triple-blinded), clinical trials, observational studies, studies conducted on post-menopausal women, studies conducted on the elderly age group, and articles published in languages other than English.

Thereby, we had a total of 10 case reports which we included in our study [28-37]. They are listed in Table 1.

**Table 1:** Records used in our study

Title	Author	DOI
Concomitant gallbladder agenesis with methimazole embryopathy	Hirotaka Kato et al. [28]	https://doi.org/10.12659/AJCR.926310
Carbimazole embryopathy: implications for the choice of antithyroid drugs in pregnancy	P. Bowman et al. [29]	https://doi.org/10.1093/qjmed/hcq248
Aplasia cutis congenita of the scalp: Therapeutic modalities	S.K. Shivakumar et al. [30]	https://doi.org/10.4103/0028-3886.27165
Postpartum hypothalamic adrenal insufficiency with remission: A rare case	Yuko Akehi et al. [31]	https://doi.org/10.1507/endocrj.EJ16-0066
A neonate with a diagnosis of pontocerebellar hypoplasia type 6 treated with biotin and developed biotin interference with laboratory thyroid function tests	Motomichi Nagafuji et al. [32]	https://doi.org/10.12659/AJCR.934417
Gastroschisis due to maternal exposure to carbimazole in a term neonate	Suzan S Asfour and Al-Mouqdad [33]	https://doi.org/10.7759/cureus.24808
A rare vascular lesion of newborn: Cutis marmorata telangiectatica congenita	Ezgi Yangın Ergon et al. [34]	https://doi.org/10.5152/TurkPediatriArs.2018.5557
Fetal hydrops, associated with maternal propylthiouracil exposure, reversed by intrauterine therapy	N. Yanai and D. Shveiky [35]	https://doi.org/10.1002/uog.977
Antenatal management of recurrent fetal goitrous hyperthyroidism associated with fetal cardiac failure in a pregnant woman with persistent high levels of thyroid-stimulating hormone receptor antibody after ablative therapy	Tadashi Matsumoto et al. [36]	https://doi.org/10.1507/endocrj.EJ13-0248
Fetal hyperthyroidism associated with maternal thyroid autoantibodies: A case report	Paraskevi Kazakou et al. [37]	https://doi.org/10.1016/j.crwh.2018.e00081

Data Analysis

Articles to be used in this literature review were finalized and data were extracted. 

The data were tabulated using Microsoft Excel and referencing was done using EndNote. Data were then analyzed by multiple authors and extrapolated into the Results section. Any conflicts were resolved via discussions with a senior author. 

Ethical approval was not required for this study as the data were obtained from already available databases and no patients were directly involved.

Results 

Demographics

The following section will present an outline of the demographic characteristics of the participants who were included in the papers analyzed for this research paper. These case reports focused on the various impacts on the fetus while the pregnant mother has hyperthyroidism. Studies comparing MMI and PTU concentrate on the age range at which these medications were administered, with primary emphasis on newborns affected by hyperthyroidism or maternal Graves' disease. To completely grasp the effects on neonatal health, it is crucial to take into account both the gestation age and the timing of the medication initiation. A total of 10 case reports were included in this literature review. The research papers were made available for public access from 2004 to 2022, covering diverse geographical areas such as the USA, the UK, Europe, and Asia. The ethnic composition of the participant groups varied across the studies. Five studies were conducted predominantly in Japanese populations, while five studies included participants from different geographical regions. In most of the studies reviewed, hyperthyroidism was detected prior to pregnancy. Only one case was detected 10 months postpartum.

Among the 10 studies reviewed, there was no specific socioeconomic status reported for the mothers. However, all 10 studies consistently showed that every mother included in the research had a history of exposure to ATDs, either before or during pregnancy. The total number of fetuses with in utero exposure to MMI, carbimazole, or PTU was 11. It is noteworthy to mention in the studies that the majority of the gestation period ranged from 36 to 38 weeks. As the majority of mothers in the case reports had been diagnosed with Graves’ disease before becoming pregnant, they were already undergoing antithyroid treatments such as PTU and MMI. Fetal hyperthyroidism seems to be consistently diagnosed in most fetuses.

**Figure 1:**
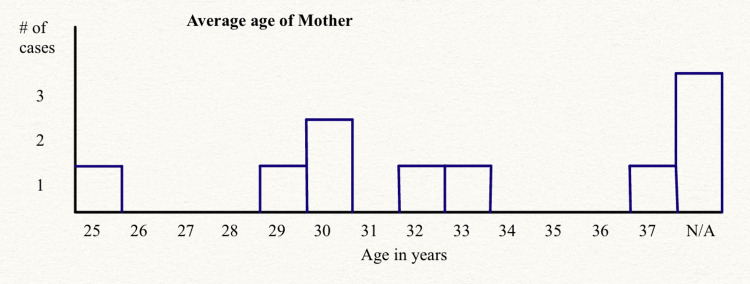
Distribution of average ages of mothers in the cases studied

The current sample size for assessing the effects is relatively small. However, by increasing the sample size, the statistical power of the findings can be enhanced, enabling a more accurate assessment of the effects of MMI and PTU in newborns and leading to more reliable conclusions. The age group of mothers included in the reviewed studies spanned from 25 to 37 years with a significant focus on individuals in their early 30s which is demonstrated in Figure 1.

In conclusion, the studies reviewed included a broad spectrum of mothers, considering factors such as age, ethnicity, geographical location, and health history. Based on the analysis of the 10 case studies, it can be concluded that the maternal utilization of PTU and MMI is linked to fetal congenital abnormalities, regardless of the trimester in which it is administered as treatment. The comprehension of the demographic characteristics of the participants is vital for interpreting the consequences of various ATDs on fetal health and could contribute to the development of effective intervention strategies. 

MMI Fetal Effects Diagnosis and Treatment of the Effect

Two out of all the studies included MMI for treating hyperthyroidism in pregnant women. One of the babies suffered from aplasia cutis, which is the absence of the scalp and calvarium. Other babies suffered from neonatal hyperthyroidism presenting with symptoms of intrauterine growth restriction (IUGR), hydrops, oligohydramnios, and goiter. The former baby was treated with MMI and propranolol and the latter with surgery and antibiotics. 

In two of the studies, the mother was treated with carbimazole. The neonate of the first mother suffered from patent vitellointestinal duct and laryngomalacia in addition to aplasia cutis. Surgery was performed to correct the patent vitellointestinal duct. The second neonate suffered from gastroschisis and subsequent intestinal malrotation. A surgical attempt was made to save the neonate but the baby succumbed to complications at 112 days.

PTU Fetal Effects Diagnosis and Treatment of the Effect

From the studies conducted which included the use of PTU for “the management of hyperthyroidism in pregnant women, the baby was reported to have varicosities on the skin of the lower extremities and back and was diagnosed with cutis marmorata telangiectasia congenita which resolved without treatment.”

Three other studies involved babies diagnosed with neonatal hyperthyroidism. The first neonate was reported to have tachycardia which was treated with MMI, levothyroxine, and atenolol. Fetal tachycardia, “and delayed developmental milestones were reported in the second neonate which was treated with MMI and levothyroxine at birth.”

Bilateral pyelectasis, vesicovaginal fistula, anal stenosis, and polydactyly were the other effects mentioned in another case report for which surgical reconstruction was done for closure of “the vesicovaginal fistula and agenesis of the vagina.”

Other Relevant Findings

Based on the studies conducted, we can examine the correlations between preterm, post-term, and normal-term delivery, as well as the correlation between the way fetal delivery was performed, based on the date of diagnosis of hyperthyroidism and treatments received before pregnancy.

Regarding the diagnosis of hyperthyroidism, out of the 11 pregnancies, almost half (5/11) had a diagnosis before pregnancy. One mother was diagnosed postpartum and another was diagnosed during pregnancy. For the remaining four mothers, the specific timing of the diagnosis was not specified. This distribution shows that hyperthyroidism can be diagnosed at various stages, either before, during, or after pregnancy.

When considering the date of fetal delivery, seven out of 11 patients delivered at term, while the remaining four mothers experienced preterm delivery. This indicates a roughly equal distribution between term and preterm deliveries among the patients included in the data.

Looking at the correlation between the diagnosis of hyperthyroidism and the method of fetal delivery, there does not appear to be a clear pattern. Among the 11 babies, four were delivered by cesarean, three by vaginal delivery, and the delivery method was not specified for four. The distribution of delivery methods is not consistent enough to establish a strong correlation between the date of hyperthyroidism diagnosis and the method of fetal delivery.

Considering the treatment received before pregnancy for hyperthyroidism, the patients were subjected to various interventions. Two patients received carbimazole, two were treated with PTU, three patients underwent levothyroxine treatment or thyroidectomy with radioiodine therapy, and two patients received MMI. Additionally, for two patients, the specific treatment administered was not specified. It is worth noting that the distribution of treatment types does not align with a particular pattern in relation to the method of fetal delivery.

Discussion

Summary

The results of the case reports discussed focus on the fetal impact of hyperthyroidism and maternal use of ATDs for the same. A total of 10 case reports have been studied, spanning various geographical areas like the UK, Europe, and Asia that included mothers between the age group of 25-37 years. Most cases consist of Graves' disease detected prior to pregnancy and reviewing the fetal effects of exposure to maternal use of ATDs like PTU, carbimazole, and MMI. The maternal use of these drugs has been linked to fetal hyperthyroidism and other congenital anomalies.

MMI use has been associated with increased cases of aplasia cutis and neonatal hyperthyroidism that are presented as IUGR, oligohydramnios, and goiter. The use of PTU has been linked with cutis marmorata telangiectasia congenita, a form of varicosities on the back and lower limbs that is self-resolving. Neonatal hyperthyroidism was treated with MMI and levothyroxine while tachycardia was stabilized using atenolol. Delayed milestones, bilateral pyelectasis, vesicovaginal fistula, anal stenosis, and polydactyly are other essential effects noted with PTU.

It is worth noting that although there was an increase in the associated fetal abnormalities after exposure to maternal use of anti-thyroid medications, there does not seem to be a clear pattern linking the diagnosis of hyperthyroidism and the method of fetal delivery or between the treatment received before pregnancy and the mode of fetal delivery.

Pathophysiology in Pregnancy 

Thyroid hormones are essential for normal fetal development. During the first trimester, the fetus depends on the maternal supply of thyroid hormones via the placenta. At 12 weeks, the fetus begins producing thyroid hormone and becomes self-sufficient at 18-20 weeks of gestation.

The maternal thyroid glands physiologically enlarge during pregnancy due to the effect of circulating maternal hCG and estrogen. Thyroid problems in pregnancy can range from maternal hyperthyroidism/subclinical hyperthyroidism/hypothyroidism/subclinical hypothyroidism. It is important to manage thyroid diseases in pregnancy to ensure favorable maternal and fetal outcomes.

Methods of treatment vary depending on the type of thyroid disorder. While iodine and thyroxine supplementation can be used to treat subclinical hypothyroidism, subclinical hyperthyroidism almost needs no treatment as it has very few clinical effects [38].

The most common cause of hyperthyroidism in pregnancy is Graves' disease and hcg-mediated hyperthyroidism. Another rare cause of hyperthyroidism in pregnancy is hyperemesis gravidarum due to excess hcg which stimulates thyroid hormone production in early pregnancy and typically subsides in the second half of pregnancy [39,40].

Maternal exposure to ATDs can have various adverse effects on the fetus. Lack of thyroid hormone in the fetus can lead to fetal hypothyroidism, intellectual problems, congenital malformations, and urogenital and musculoskeletal problems.

Exposure to low thyroid levels can stimulate the fetal pituitary, causing high TSH levels. Chronically elevated TSH levels make way for goiter, which can cause dystocia and airway obstruction in a neonate.

Hydrops fetalis is also another manifestation of fetal hypothyroidism. ATDs include PTU and MMI.

PTU is considered safer for use in pregnancy. PTU and MMI inhibit thyroid hormone production by blocking thyroid peroxidase, which aids in the oxidation of iodide. This interrupts the organification of iodine, which leads to the inhibition of thyroid hormone synthesis. PTU also has peripheral activity and inhibits the conversion of T4 to T3 by blocking the enzyme 5'-deiodinase [35].

Although both drugs have been used in pregnancy, PTU is considered safer but there is no evidence that favors one drug over the other. No evidence of a difference in the incidence of congenital malformations between the two drugs has been reported. Certain studies have shown an increased number of cases of aplasia cutis congenita with the use of MMI. However, infants exposed to maternal ATDs in utero do not show long-term cognitive problems. Exposure to maternal PTU and MMI has been associated with fetal congenital abnormalities, muscular hypotonia, atresia of the aorta, syndactyly, and congenital dislocation of the hips. Fetal thyroid status is evaluated using cordocentesis. Treatment of fetal goiter and high-output cardiac failure due to shunting can be treated effectively with intra-amniotic thyroid therapy.

Current Guidelines

TSH levels are low throughout the first trimester of pregnancy and rise afterward. The American Thyroid Association (ATA) and the Endocrinology Society both recommend that the maximum limit for TSH be 2.5 mlU/L in the first trimester, 3.0 mlU/L in the second trimester, and 3.5 mlU/L in the third trimester based on the most recent studies. The first trimester's lower limits are 0.1 mlU/L, the second trimester's lower limits are 0.2 mlU/L, and the third trimester's lower limits are 0.3 mlU/L [39].

For pregnant women, the World Health Organisation (WHO) and the US Institute of Medicine (IOM) recommend a daily iodine intake of 220-250 mcg. Most studies suggest testing urine iodine content, with typical values between 150 and 249 g/L, as a way to evaluate iodine consumption [39].

The examined recommendations all advocate focused screening of high-risk patients by assessing blood TSH levels rather than general testing for thyroid function disorders before and throughout pregnancy. Additionally, they all stress the importance of treating overt hypothyroidism and hyperthyroidism, not just during pregnancy but also prior to conception, and they all advise similar management strategies and treatment objectives. There is also unanimity on how to handle cases of probable fetal thyrotoxicosis, postpartum thyroiditis, and thyroid cancer as well as gestational transitory hyperthyroidism with hyperemesis gravidarum. It is not advised to use radioactive iodine for scanning or treatment when pregnant or nursing [41].

In addition to the patient's age, the presence of risk factors, and symptoms, proposed grading systems discriminate between mild (TSH, 0.1-0.4 mIU/L) and severe subclinical hyperthyroidism (TSH, 0.1 mIU/L). These characteristics are used to guide treatment. An appropriate examination will look into the underlying reason and evaluate a person's risk factors to decide whether therapy is necessary and what kind may be suggested [42].

Treatment of Graves' Hyperthyroidism During Pregnancy 

Antithyroid medications, especially PTU, are the first line of treatment for Graves' illness during pregnancy. 

To keep maternal serum-free T4 levels at or slightly over the top limit of the normal non-pregnant range or total T4 levels at 1.5 times the usual non-pregnant reference range, prescribed doses should be as low as possible.

Second-trimester thyroidectomy can be considered if continuing ATD delivery is not practicable; patients should receive adrenergic blockade and iodide therapy prior to surgery.

To assess the risk for fetal hyperthyroidism, all women with current Graves' disease and levothyroxine-replaced patients with a history of I-131 mediated ablation or thyroidectomy should have their anti-TSH-receptor antibody (TRAb) levels evaluated at 26-28 weeks of gestation.

If there is an indication of active maternal Graves' illness (elevated maternal TRAb levels or maternal need for antithyroid medication), fetal ultrasonography should be done at 28-32 weeks to evaluate fetal thyroid function.

The fetal cord blood should be tested for serum TSH and total or free T4 concentrations at delivery in women with active Graves' illness or positive TRAb screens following 131I-mediated ablation or thyroidectomy [43].

Side Effects of MMI and PTU

The two main drugs that have been used, by and large, for the management of various forms of hyperthyroidism are MMI and PTU [15]. The primary mechanism of action of these drugs is to block thyroid hormone production [44]. The inhibitory effects of thioureas/thiouracils on thyroid function were first documented in animal studies. Soon after that, the US FDA issued approval for PTU, MMI, and carbimazole in the treatment of hyperthyroidism in humans. These drugs have been used abundantly over the past eight decades. However, there are no controlled data on pregnancy [44]. Fetal harm is reported when these drugs are administered to a pregnant woman. Since studies show that MMI causes more harm than PTU, its use in pregnancy has been controversial over the past few years [15].

PTU is the drug of choice for the treatment of hyperthyroidism in pre-pregnancy as well as the first trimester of pregnancy, or even up to 16 weeks of gestation [45], but a switch to MMI is preferred during the second and third trimesters because PTU is highly hepatotoxic. MMI causes fewer side effects, and these are generally dose-related [46]. A comparison of both is shown in Table 2.

**Table 2:** Comparison of the side effects of propylthiouracil vs methimazole SJS, Stevens-Johnson syndrome; TENS, toxic epidermal necrolysis; AKI, acute kidney injury.

Propylthiouracil [45]	Methimazole [15]
Hepatotoxicity	Agranulocytosis
Hypersensitivity	Hypersensitivity
ANCA-associated vasculitis	Hepatotoxicity
Agranulocytosi	Hypothyroidism
Skin rash, SJS, TENS	Hyperpigmentation
AKI, acute interstitial nephritis	Cross reaction with beta-blockers and digoxin
Loss of taste, nausea, vomiting	Nausea, vomiting
Neuritis, headache, vertigo	
Lupus-like syndrome	
Drug fever	
Hypoprothrombinemia	
Hypothyroidism	

Hepatotoxicity of PTU

Hepatotoxicity is the most dreaded and most observed complication of PTU. Therefore, laboratory monitoring of thyroid function tests with TSH and free T4 every 2-4 weeks initially and then 4-6 weekly once thyroid hormone levels are stabilized is essential while the patient is on PTU therapy [47].

Agranulocytosis of MMI

It is defined as having an absolute granulocyte count of less than 500/mL. It most commonly occurs within the first three months of beginning therapy. The most common symptoms of agranulocytosis are fever and a sore throat. Patients should be advised to get a white blood cell count as soon as the symptoms appear. Intravenous antibiotics should be used to treat fever or any obvious infections. Since this complication has been noted with PTU as well, avoid the drug in patients who develop agranulocytosis with MMI.

MMI is contraindicated if there is hypersensitivity to the drug or any of its components. It is relatively contraindicated during pregnancy especially in the first trimester, except in patients who cannot tolerate PTU. Usually, the lowest effective doses are started. Patients receiving MMI should be constantly followed and advised to report any signs of illness. Acquire total and differential cell counts and search for signs of agranulocytosis. MMI and PTU have been linked to hypoprothrombinemia and hemorrhage. Prothrombin time should be monitored in such patients, especially before surgery. A dose reduction or discontinuation of therapy may be possible, several weeks or months before delivery. Beta-blocker clearance might be increased by hyperthyroidism. As a result, when a patient with hyperthyroidism achieves euthyroid status, the dose of beta-blockers may need to be reduced. When a patient with hyperthyroidism achieves euthyroid status, the dose of digoxin may need to be reduced. When hyperthyroid individuals on a stable theophylline regimen become euthyroid, theophylline levels rise; a lower dose of theophylline may be required [15,46].

The major hindrance to the use of PTU is the fact that it has lesser efficacy than MMI, which is taken as a once-a-day tablet, compared to PTU which requires 2-3 doses a day, resulting in lesser compliance. A clear demonstration of the teratogenic effects of MMI is currently not present. Therefore, it seems reasonable to follow the current guidelines and advice for PTU treatment in hyperthyroid women during the first trimester of pregnancy. Large and prospective worldwide studies will be needed to fully clarify the issue of ATD safety during pregnancy. In the absence of a compelling indication for the use of MMI, PTU should still be considered as the first-line agent in the treatment of Graves' disease during early pregnancy. MMI should be considered as a viable second choice if the patient cannot tolerate PTU, has an allergic reaction to PTU, or does not become euthyroid while receiving PTU even during the first trimester, since studies have shown that the incidence of congenital malformations is greater in babies of mothers whose hyperthyroidism has remained untreated than in those who have been treated with antithyroid medications [47,48].

Why MMI is still not preferred in the first trimester of pregnancy is because of its highly teratogenic nature. MMI can easily pass the placental membrane due to its low protein binding and produces severe deleterious effects on the fetus throughout the organogenesis phase, especially when taken during the first trimester, whereas effects exerted by PTU are less severe and non-fatal [15]. These are compared in Table 3.

**Table 3:** A comparison of the fetal effects of methimazole and propylthiouracil

Methimazole [46,49]	Propylthiouracil [45]
Goiter, cretinism, aplasia cutis, umbilical anomalies, facial dysmorphism, esophageal atresia, craniofacial malformations, choanal atresia, omphalocele, tracheoesophageal fistula, developmental delay, renal, skull, cardiovascular, congenital defects, gastrointestinal malformation, duodenal atresia	Birth defects of the face, neck, and urinary system, fetal hypothyroidism (rare), and neurodevelopmental delay (rare)

Although MMI is teratogenic, there is no scientific study that shows that there is any associated risk of preterm birth or low birth weight in babies of pregnant women taking MMI for hyperthyroidism. Also, there is no study that shows that MMI causes stillbirths or miscarriages. Another study suggests that the gestational age at delivery and the rate of vaginal delivery did not significantly differ from pregnant women not taking MMI [48,50].

PTU and MMI are secreted in low concentrations in breast milk but have no effect on the infant's thyroid function, and breastfeeding is permitted at moderate doses of these drugs. When compared to alternative treatments, several specialists advocate MMI as the antithyroid medicine of choice for nursing mothers. With a maximum daily dose of 20 mg, maternal MMI use has no effect on intellectual development or thyroid function in breastfed infants whereas mothers can breastfeed while taking PTU at doses as high as 750 mg daily without adverse effects on thyroid status in their infants. To reduce newborn exposure, recommend that the patient take MMI immediately after nursing or wait 3-4 hours before nursing. Features of fetal thyroid dysfunction on ultrasound include growth limitation, advanced bone age, goiter, or heart failure. The infant should be monitored for evidence of infection, as unusual reactions (e.g., agranulocytosis) may occur. In addition, if a drug-induced blood dyscrasia is suspected, keep an eye on the infant's complete blood count and differential [15,51-54].

Healthcare outcomes on PTU therapy can be improved by an interprofessional team approach which involves the collaborative efforts of clinicians (MDs, DOs, NPs, and PAs), specialists, specialty-trained nurses, and pharmacists, working together across disciplines to achieve the wanted patient results while also minimizing adverse events. It is also just as important to counsel patients regarding the possible side effects and educate them regarding the importance of contacting the physician if they are on PTU or MMI therapy and become pregnant or intend to become pregnant for proper dose adjustment and monitoring throughout the pregnancy. In case any of the members of this interprofessional team identifies an adverse event, drug interaction, or even therapeutic failure in a patient who is on PTU or MMI therapy, the event should be reported to the other team members, after proper documentation in the patient’s records, so that it can be analyzed [45].

Other Treatments Available

Other than oral medications, there is also surgery and radioactive iodine. Radioactive iodine is used in a way that radiotherapy can be concentrated only in the cells that use iodine, which are the thyroid cells. It will destroy the thyroid cells, and is a very effective treatment, especially for Graves’ disease or when the antithyroid medicines are not working. This is contraindicated in pregnant women, so it would not work as a choice of treatment in this case. Surgery is used when major secondary effects happen when people are taking ATDs. It has also been a preferred method for pregnant women. Usually, a complete thyroidectomy is not done, just a part of the thyroid is taken away [3]. 

Long-Term Effects If Not Treated 

Untreated subclinical hyperthyroidism during pregnancy may affect the mother in the long term, but they are not as harmful as in women with a more advanced pathology [55]. 

Previous studies also demonstrate that it is unlikely for women to develop any problems during pregnancy, although it is recommended that it is treated. If left untreated, adverse effects in both the mother and the infant are common [56].

As thyroid hormones can cross the placenta, the fetus may develop a thyroid pathology when born. The baby may also be born underweight, with mental retardation, or even premature. In the worst case, it may lead to death [57]. The mother, in the long term, can develop a heart disease caused by having high levels of T4 for a long time. It can also lead to hemorrhage posterior to the placenta [58].

Limitations

It is important to acknowledge the constraints that come with doing a literature study. Keeping these hurdles in mind, we can ensure that this knowledge gap is seen through. The caliber of the various included studies can considerably impact the validity and generalizability of a literature review. Limitations can be introduced by studies with small sample sizes or biased data-gathering methods, which can also have an impact on the review's overall quality. Drawing firm conclusions might also be hampered by the diversity in research designs, participants, and settings across studies. Literature reviews rely on previous studies published on a certain date and are subject to temporal restrictions. As a result, recent research or new trends might not be included, thereby reducing the review's accuracy and applicability.

The lack of studies explicitly concentrating on pregnant women treated with PTU is another major shortcoming of literature evaluations in the area of hyperthyroidism during pregnancy. This restriction exists due to various reasons. First, ethical concerns frequently forbid conducting prolonged studies on pregnant women beyond the first trimester, which may limit the number of currently available studies. Secondly, the prevalence of hyperthyroidism during pregnancy is comparatively low. As a result, it is possible to be limited to theoretical foundations when discussing the conclusions on the efficacy and safety profile of PTU in expectant women.

Prospectively, this limitation can be resolved by more research conducted by organizations, physicians as well as researchers. Studies targeted at this particular demographic must be carried out for a better understanding of the pathophysiology of subclinical hyperthyroidism that can later help guide treatment strategies.

## Conclusions

Based on the results of the studies mentioned above, there is sufficient proof to suggest that the use of MMI and carbimazole to treat hyperthyroidism during pregnancy may have adverse effects on the fetus that is still developing. The detrimental sequelae include aplasia cutis, and neonatal hyperthyroidism with signs of IUGR, hydrops, oligohydramnios, and goiter. Congenital defects, specifically patent vitellointestinal duct, laryngomalacia, gastroschisis, and intestinal malrotation, were observed. It is essential to remember that while some reported issues were effectively dealt with, others had unfavorable effects.

In conclusion, the review of the data that is accessible fails to demonstrate a conclusive association between the onset of hyperthyroidism diagnosis (before, during, or after pregnancy) and the term of the fetal delivery (preterm, post-term, or term). The prevalence of patients in these categories appears to be varied, indicating that hyperthyroidism can be diagnosed at any time and that the approach of management is not always determined by the date of the diagnosis. Similarly to this, there is no apparent correlation between the mode of fetal delivery and the specific therapies for hyperthyroidism received before becoming pregnant.

It is important to note that this analysis only includes a small number of studies and that a very limited number of cases were reported for each treatment strategy. This restricts the findings' capacity to be generalized and emphasizes the need for more study in this area. The studies also included some instances where the neonates did not show serious ailments or when the stated illnesses were resolved without intervention. 
